# Can primary care team-based transition to insulin improve outcomes in adults with type 2 diabetes: the stepping up to insulin cluster randomized controlled trial protocol

**DOI:** 10.1186/1748-5908-9-20

**Published:** 2014-02-14

**Authors:** John S Furler, Doris Young, James Best, Elizabeth Patterson, David O’Neal, Danny Liew, Jane Speight, Leonie Segal, Carl May, Jo-Anne Manski-Nankervis, Elizabeth Holmes-Truscott, Louise Ginnivan, Irene D Blackberry

**Affiliations:** 1 General Practice and Primary Healthcare Academic Center, University of Melbourne, 200 Berkeley St, Carlton, VIC 3053, Australia; 2 School of Medicine, University of Melbourne, Parkville, Australia; 3 School of Nursing, University of Melbourne, Parkville, Australia; 4 Department of Medicine, St Vincent’s Hospital, University of Melbourne, Fitzroy, Australia; 5 Melbourne EpiCenter, the University of Melbourne, c/-The Royal Melbourne Hospital, 7 East, Main Building, Grattan St, Parkville, VIC 3050, Australia; 6 The Australian Center for Behavioural Research in Diabetes, Diabetes Australia – Victoria, Melbourne, Australia; 7 Center for Mental Health and Wellbeing Research, School of PsychologyDeakin University, Burwood, VIC Australia; 8 AHP Research, Hornchurch, Essex, UK; 9 School of Population Health Division of Health Sciences, University of South Australia, Playford Building P4-26, City East Campus, CEA-24, North Terrace, Adelaide SA 5000, Australia; 10 Faculty of Health Sciences, University of Southampton, Highfield, Southampton SO17 1BJ, UK

**Keywords:** Type 2 diabetes, Primary care, Nursing, Randomized trial, Insulin, Implementation, Australia, Health services research

## Abstract

**Background:**

Type 2 diabetes (T2D) brings significant human and healthcare costs. Its progressive nature means achieving normoglycaemia is increasingly difficult, yet critical to avoiding long term vascular complications. Nearly one-half of people with T2D have glycaemic levels out of target. Insulin is effective in achieving glycaemic targets, yet initiation of insulin is often delayed, particularly in primary care. Given limited access to specialist resources and the size of the diabetes epidemic, primary care is where insulin initiation must become part of routine practice. This would also support integrated holistic care for people with diabetes. Our Stepping Up Program is based on a general practitioner (GP) and practice nurse (PN) model of care supported appropriately by endocrinologists and credentialed diabetes educator-registered nurses. Pilot work suggests the model facilitates integration of the technical work of insulin initiation within ongoing generalist care.

**Methods:**

This protocol is for a cluster randomized controlled trial to examine the effectiveness of the Stepping Up Program to enhance the role of the GP-PN team in initiating insulin and improving glycaemic outcomes for people with T2D. 224 patients between the ages of 18 and 80 years with T2D, on two or more oral hypoglycaemic agents and with an HbA1c ≥7.5% in the last six months will be recruited from 74 general practices. The unit of randomization is the practice.

Primary outcome is change in glycated haemoglobin HbA1c (measured as a continuous variable). We hypothesize that the intervention arm will achieve an absolute HbA1c mean difference of 0.5% lower than control group at 12 months follow up. Secondary outcomes include the number of participants who successfully transfer to insulin and the proportion who achieve HbA1c measurement of <7.0%. We will also collect data on patient psychosocial outcomes and healthcare utilization and costs.

**Discussion:**

The study is a pragmatic translational study with important potential implications for people with T2D, healthcare professionals and funders of healthcare though making better use of scarce healthcare resources, improving timely access to therapy that can improve disease outcomes.

**Trial registration:**

Australian and New Zealand Clinical Trials Registry ACTRN12612001028897

## Background

Type 2 diabetes (T2D) is a costly, epidemic condition [1,2]. In Australia, T2D is anticipated to be the leading cause of disease burden by 2016 [2], costing over $14 billion annually in direct and indirect healthcare costs [3], and shortening life expectancy by up to five years. We can expect an exponential rise in the human and healthcare costs associated with T2D [4].

Because T2D is a progressive condition, involving beta cell failure in the presence of insulin resistance, normoglycaemia is increasingly difficult to achieve [5]. Hyperglycaemia causes microvascular and macrovascular complications through glycation and oxidation of proteins [6]. Conversely, achieving optimal glycaemic control early in the course of the disease reduces the risk of vascular complications as well as mortality [7]. The most recent Australian Diabetes Society (ADS) guidelines [8] recommend individualized glycaemic targets (HbA1c) based upon diabetes duration, age, and co-morbidity, yet in Australia, only 52% of people with T2D achieve an HbA1c below 7% (53 mmol/mol).

Early use of insulin as a part of treatment intensification for people with T2D is supported by both American and European guidelines [9-11]. The effectiveness of insulin in improving glycaemic control in T2D has been shown in clinical trials, largely in secondary care [12-15]. The use of long-acting insulin analogues with simple patient-driven algorithms is feasible, safe (low incidence of hypoglycaemia), effective in achieving glycaemic control [13,14], and associated with improved patient satisfaction [16].

Despite evidence of the effectiveness of insulin and endorsement of its early use in guidelines, in practice, initiation of insulin is often delayed. The mean HbA1c of people with T2D prior to starting insulin is 9.4% (79.2 mmol/mol) in Australia (after a median diabetes duration of 8.1 years) [17], and 9.3% (78.1mmol/mol) in the UK [18], well above recommended targets. This has been attributed, in part, to ‘psychological insulin resistance’ (negative perceptions and attitudes that act as barriers to starting insulin) [19] among people with T2D and ‘clinical inertia’ (recognition of a problem but failure to act) among practitioners [20]. However, health system factors also play a role. While the majority of care for people with T2D takes place in general practice, insulin initiation is commonly deferred to specialist diabetes service [21,22], and the majority of Australians with T2D initiate insulin within specialist settings [23]. Difficulties accessing endocrinologists and diabetes nurse educators because of cost and limited availability can lead to delays in starting insulin, and avoidable periods of hyperglycaemia.

While specialist diabetes services have a role in managing complex cases, initiating insulin in routine general practice has several benefits. For example, diabetes care can remain integrated with care for other conditions, as glycaemic control is often just one facet of managing multiple co-morbidities. Furthermore, as secondary care is costly [24], there are considerable economic benefits to retaining T2D care in general practice. However, achieving timely and effective insulin initiation as a routine aspect of care in general practice presents some challenges. GPs are more reluctant than specialists to start insulin [25], and up-titrating to achieve glycaemic targets is also a challenge. A Canadian trial utilising community pharmacists and specialist nurses to support general practice-based insulin initiation did not show any improvement in insulin prescribing rates or in glycaemia [26]. An intensive educational intervention for GPs and PNs in the UK has been evaluated using clinical audit data from 115 practices; it showed significantly improved glycaemic control at six months in patients who started insulin, although further improvement was not observed over 36 months [27]. In Australia, 63% of general practices employ close to eleven thousand PNs across the country [28]. They are a rapidly growing sector of the primary care workforce and now core members of the general practice team supported by significant government investment.

We have designed and piloted for feasibility [29] an education and system-change intervention (the Stepping Up Program—described in more detail below) to facilitate general practice-based insulin initiation in people with T2D and suboptimal glycaemic outcomes who are on maximum oral therapy. The program is based on a model of care that focuses on the ‘in-practice team’ (GP and PN), supported appropriately by endocrinologists and credentialed diabetes educator-registered nurses (CDE-RNs). Our model acknowledges the importance of endocrinologists and CDE-RNs in providing support to GP/PN teams in initiating insulin when needed. Our pilot work [29] highlighted how this model of care supported integration of the technical work of insulin initiation within ongoing generalist care. Issues of clinical accountability and flexibility, managing time and resources were highlighted as important.

In this paper, we describe the protocol for a cluster randomized controlled trial to examine the effectiveness of the Stepping Up Program to enhance the role of the GP-PN team in initiating insulin and improving glycaemic outcomes for people with T2D. Our primary outcome is change in HbA1c (measured as a continuous variable). We hypothesize that participants in the intervention arm will achieve an absolute HbA1c mean difference of 0.5% lower than control group participants at 12 months follow up. Secondary outcomes are described below.

## Methods

This study is a cluster randomized controlled trial, conducted in 74 general practices across the Australian state of Victoria. The consort diagram is provided in Additional file 1. Ethical approval was received from the University of Melbourne Health Sciences Human Research Ethics Sub-committee (ID 123740). The trial is registered with the Australian and New Zealand Clinical Trials Registry (ACTRN12612001028897).

### Recruitment and randomization of practices

Eligible general practices must have a current PN. Practices are recruited using our University Department of General Practice database (the VicREN practice based Research Network) and through Medicare Locals. Practice randomization is stratified by practice size, location in Community Health Centers, and participation in the National Primary Care Collaboratives.

The unit of randomization is the practice. Practices are randomized after consenting, and following recruitment of at least one eligible patient (Additional file 1). Due to the nature of the intervention and because further patient recruitment often occurs over a period of months subsequent to randomization, full allocation concealment is not possible. Randomization is in blocks, undertaken by a statistician at the University of Melbourne independent of the research team. Following randomization, the research team assist practices to continue to identify and recruit patients.

### Participant eligibility

Eligible patients are those with most recent HbA1c (in the previous six months) ≥7.5% and who are on maximal oral therapy but not currently on insulin. Maximal oral therapy is defined as using at least two oral hypoglycaemic agents (OHAs) (metformin, sulphonylurea, TZDs, DPP-4 inhibitor) at maximal tolerated doses. Congruent with the pragmatic, translational nature of this trial, patients are also eligible if their responsible medical practitioner is of the opinion that insulin is appropriate despite the patient being on less than two OHAs or not at maximal dose. Patients are ineligible if they are >80 years old, have unstable cardiovascular disease, are unable to give informed consent or have a complex debilitating medical condition, *e.g.*, severe mental illness, end-stage cancer.

### Participant identification and recruitment

Patient identification is conducted by practices employing a number of methods to generate a comprehensive list of potentially eligible patients. Search methods vary by practice but include searching the practice electronic medical records database, using the Pen clinical audit tool [30], or obtaining a list of HbA1c results from the local pathology providers. Our previous research and pilot suggests an average general practice (of two full-time GPs) has 80 to 100 identified patients with T2D, 10% to 15% (8 to 15) of whom meet inclusion criteria.

Recruitment involves a letter from the practice inviting eligible patients to participate and a follow-up call from the PN and/or GP. The invitation letter states that participants may benefit from assessment and more intensive treatment of their diabetes and that insulin may be a part of that program. Patients are invited to express interest in receiving further details about the study, to attend the practice to learn more about the study from a member of the study team. If the patient agrees to participate, consent and baseline data are collected at that visit. Patients are asked to have their HbA1c checked and are subsequently excluded if the result is <7.5%.

### Study intervention

Our intervention is targeted at the decision to initiate insulin and the care processes involved in implementing that decision. The Stepping Up Program intervention (Additional file 2) is designed to address barriers we have identified. GPs and PNs in intervention practices participate in the Stepping Up Program elements: in-practice briefing and training visit, and ongoing support for practice and PN in working with patients.

The aim of the training is to ensure GP/PN teams have the knowledge and confidence to initiate a discussion with eligible patients about commencing insulin, knowing that they will be able to act on this within current routine clinical care using a simple, safe, convenient evidence-based algorithm. Referral or consultation with an Endocrinologist, CDE-RN or any other appropriate health professional will naturally be management options for GPs managing study patients. GPs and PNs are provided with patient packs for each of the participating patients whom they review.

In control group practices, GPs are sent a copy of Royal Australian College of General Practitioners (RACGP) guidelines for the management of T2D and a list of participating patients and asked to undertake a clinical review of those patients. Control group practices will be offered the Stepping Up training after the 12-month follow up.

Following recruitment and baseline data collection, participants are invited to attend their GP for an assessment to discuss their diabetes. For the first participant this is at a time when the Study CDE-RN is available to support the PN. Participants in intervention practices are managed according to the Stepping Up Program by GP and PN who have attended the Stepping Up Training. When participants attend, the GP reviews their diabetes and if appropriate makes a clear recommendation for the need to commence insulin, refers the participant to the PN and writes a prescription for Glargine Insulin. The participant sees the PN (supported by the Study CDE-RN) on that day for an insulin initiation assessment, during which time the PN and the Study CDE-RN work through the patient pack with the participant. At that visit the PN, supported by the CDE-RN, gives the first dose of Glargine insulin 10 units. The PN has the option to review the participant on day two, and observe the participant give their second dose of Glargine 10 units to themselves if they are not already comfortable to do this at home. Subsequently participants are asked to call the PN for review by phone every three days. The participant records fasting morning blood glucose in the record book provided and is encouraged to discuss the results with the PN and follow the simple protocol (see Additional file 3). After four weeks, if fasting blood glucose levels (FBGLs) are at target (<7), participants are reviewed by the GP and a three-day blood glucose level (BGL) profile is completed to identify the meal with the largest postprandial (PP) excursion. Apidra is then commenced at this meal and again adjusted to a simple protocol based on an average PPBGL over three days related to that mealtime (see Additional file 3). GPs review participating participants at least every four weeks. The GPs initiate and prescribe insulin, while the insulin adjustment is led primarily by PN and participant in discussion and in liaison with the GP as necessary.

All participants, even if not commencing insulin at the first GP/PN visit, continue to see the PN and GP with the aim of commencing insulin. GPs and PNs see participants on as many occasions as is clinically necessary over a period of up to 12 months drawing as needed on the Stepping Up Program manual and resources.

Participants in control practices are managed by their GP according to usual care (*e.g.*, referral, investigation, and adjustment of medication as the GP believes clinically appropriate).

### Specific role of the practice nurse

PNs lead and drive the dialogue with participants around the issue of intensifying treatment through insulin initiation. PNs do not prescribe or manage dosing of insulin without liaison with the GP. Our training directly addresses any GP or PN reluctance to initiate insulin, as well as developing in-practice systems to support the initiation process. This process is well within the legal scope of practice for PNs. Any clinical concerns beyond the conservative simple clinical protocol can be addressed through consultation with an endocrinologist or local CDE-RN in the usual manner. Ongoing care of complex and co-morbid conditions continues as per usual care.

### Data collection and outcomes measures

#### Outcomes

The primary outcome is change in HbA1c (measured as a continuous variable). Secondary outcome measures include the number of participants who successfully transfer to insulin and the proportion of participants who achieve HbA1c measurement of <7.0%. We will also collect data on other outcome measures of interest including the proportion of participants who achieve individualized HbA1c targets according to Australian Diabetes Society guidelines, changes in psychometric scores including AQoL-8D, PHQ-9 and PAID and net healthcare utilization and costs. Research assistant (RA) staff will collect participant data at baseline and at 12 months using the instruments and measures outlined in Table 1. HbA1c is also collected at six months.

**Table 1:** Outcome measures and collection time points

**Measure**	**Baseline**	**6 months**	**12 months**
**Demographic and clinical measures:**			
Demographic data, duration of diabetes	X		
Biometric measures: weight, BMI, waist circumference	X		X
HbA1c	X	X	X
Lipids, U&E, spot urine albumin to creatinine ratio	X		X
Medication details, co-morbidities	X		X
**Psychological measures:**	X		X
Health status: AQoL-8D [31]	X		X
Depressive symptoms: PHQ-9 [32]	X		X
Diabetes-related distress: PAID [33]	X		X
Medication taking behaviours: MARS [34]	X		X
Beliefs about insulin: ITAS [35], additional single items taken from [36]	X		X
Patient experience: excerpts from GPAQ [37], Consumers Perception of Informational Continuity [38]	X		X

All participants (intervention and control) are provided with a BGL meter (Performa Nano™; Roche Diagnostics) and are instructed how to use the meter. Data from BGL meter are uploaded at each practice visit and at 12 months by PNs to a secure server.

A pre-printed pathology form (HbA1c) with participant and practice details entered is mailed out to study participants by the study RA five months after the baseline visit with the GP at which anthropometric data was collected. HbA1c results five to seven months from the baseline date will be accepted as the mid-study HbA1c.

Additional data will be collected to inform the economic evaluation: resources used to administer the intervention; subsequent changes in participant’s health service utilization during the 12-month follow-up period; and use and cost of medical services (data gathered by accessing health service and pharmaceutical data from Medicare Australia).

A practice characteristics survey is also collected at baseline. In addition, participating GPs and PNs complete a questionnaire that includes demographics, current perceptions and practices regarding insulin initiation, questions relating to insulin initiation and perceived participant willingness from the Diabetes Attitudes, Wishes, and Needs (DAWN) study [39], relational coordination [40], collaborative practice scale [41] and questions based on normalization process theory [42], and the interactional determinants of collaboration [43].

### Analysis

#### Power calculation and sample size

Our sample size calculation is based on the primary outcome. A total of 224 participants (average of three participants per practice) from 74 general practices are required to detect an absolute 0.5% mean HbA1c difference over 12 months between control and intervention groups with 80% power and a standard deviation (SD) of 1 using two-sided alpha of 0.05. Sample size calculation allows for a design effect of 1.05 and is based on a conservative estimate of the intra-cluster correlation (ICC) for HbA1c of 0.05 [44] and attrition rate of 10% for practices and participants.

In a community observational study [23], over a five-year period, about 2% of the community sample changed from oral treatment to insulin per year. We conservatively assume a background rate 10 times this or 20% per year in the control group. Based on our pilot study [29], we estimate 60% will start insulin in the intervention group. A total of 224 participants from 74 general practices will provide sufficient power to detect a 40% change in the proportion that transition to insulin (power = 99.9%, assuming an intra-cluster correlation of 0.3). We will measure and compare the proportion of participants in each group that reach HbA1c target although our sample size is not powered to detect a significant difference in that.

The study will also have at least 80% power to detect an effect size of 0.5 of 1 SD for psychosocial scores, assuming an ICC of 0.05.

We do not anticipate a significant confounding of the results by the attention received by control group participants. In control group practices, GPs will be advised to undertake a clinical review of participating participants. In these practices, the PN will not receive training or support. In our intervention the PN once trained plays a critical enabling role in facilitating a simple in-practice system for insulin initiation. Without access to a trained PN and this system, background rates of insulin initiation by GPs in the control arm are likely to approximate those quoted above.

#### Statistical analysis

Results will be reported according to CONSORT guidelines for cluster randomized trials. A purposely built survey has been developed. Surveys will be checked for completeness prior to being scanned and sent for processing with a data management service. Pathology data will be extracted by the study pathology provider. Data will be uploaded into Stata 12 for further cleaning and analysis.

Descriptive statistics will be used to summarize GP, PN, and participant factors for the two study groups and to check for any imbalance in potential confounders. The individual participant will be the unit of analysis and the analytical methods will allow for clustering of individuals within the practice. Marginal logistic model using generalized estimating equations with robust standard errors will be used to compare binary outcomes between the two study arms. Mixed-effects linear regression will be used to compare the means between study arms for continuous outcomes, adjusting for baseline outcome measure. Stratification variables will be included as fixed effects. Analysis will be conducted on an intention to treat basis, with any imbalance of confounders between study groups adjusted for in the regression analysis.

#### Economic evaluation

Medicare Benefits Scheme (MBS) and Pharmaceutical Benefits Scheme (PBS) data between 1 October 2011 and 1 February 2016 will be obtained to examine health services utilization prior, during, and after study period. The submission was approved by Medicare Australia in October 2012 (reference 2012/CO11645). An additional Medicare consent will be sought from each participant using the Medicare Australia consent form. Data extraction will occur in May 2016. Furthermore, a submission will be made to the Victorian Department of Health to obtain data linkage on the Victorian Admitted Episodes Datasets (VAED) and the Victorian Emergency Minimum Datasets (VEMD).

A within-trial Cost-utility analysis (CUA) will be undertaken from the perspective of the healthcare system. The primary effectiveness outcome, quality-adjusted life years, will be calculated from the AQOL-8D. We will calculate the cost of the Stepping Up Program, distinguishing implementation from establishment and trial-specific costs. The mean per participant cost of healthcare will be estimated for both intervention and control clients, based on the audit of clinical records, participant questionnaire and data linkage, as described above. The intervention group will incur the additional costs of the Stepping Up Program. One-way sensitivity analyses will be undertaken to identify the key cost and outcome drivers. A stratified analysis will be used for subgroups where the Stepping Up Program may have any greater or lesser effectiveness, defined *a priori*.

### Trial status

Baseline data collection is ongoing and we anticipate will be completed in February 214. Follow up data collection should be completed by mid-2016. Data cleaning or analysis has not yet begun.

## Discussion

The Stepping Up trial has been designed as a translational study, testing the implementation of a known efficacious intervention (insulin for treating hyperglycemia) into real world practice in primary care. One of the study’s key strength is that it is a pragmatic trial examining the real world effectiveness of reorienting care systems and the use of existing human healthcare resources to address a persistent clinical problem. It has important potential implications for people with T2D, healthcare professionals, and funders of healthcare though making better use of scarce healthcare resources, improving timely access to therapy that can improve disease outcomes.

If successful, it will inform chronic disease and diabetes routine care, policy, and funding. PNs are the fastest growing section of the primary care workforce. Almost 60% of practices employ at least one PN [28], and as core members of general practice teams, they are increasingly important in addressing the epidemic of chronic disease [45], attracting significant government interest and investment. PNs potentially have a much broader and more autonomous role than traditional clinic nurses, yet overall have had little formal training in such a role [46]. Prior research has shown that people with T2D are receptive to enhanced PN involvement in their care [47], and that PNs are keen to enhance their role within diabetes care. PNs are often able to offer the time, support, and care required to respond to the psychosocial and emotional needs of people with T2D [48]. However, local practice context and culture issues need to be addressed [49]. The Stepping Up intervention supports the GP/PN team by clarifying roles, work relationships and responsibilities.

The intervention has the potential to demonstrate significant improvements in biochemical and clinical markers known to be associated with improved outcomes in people with T2D. The study will also provide important evidence about the impact on psychosocial outcomes for people with T2D participating in the model of care and for those commencing insulin therapy in primary care.

A cost analysis of the Stepping Up program and its impact on disease progression and downstream costs within in Australia will have local relevance. This study will investigate how the costs of diabetes care are affected by better integration of PN, GP, endocrinologist and CDE-RN in the general practice setting, based on real-world implementation of the stepping Up program taking into account the support required to initiate and sustain change in practice. The expectation is of downstream cost savings through reduction of future diabetes-related complications.

Our proposed study also directly contributes to building a research culture in Australian general practice. The practices (GPs and PNs) involved in Stepping Up will be supporting part of a state wide practice-based research network to enhance primary care research capacity in Australia.

## Abbreviations

T2D: Type 2 diabetes; RA: Research assistant; SET: Screening eligibility tool; PLS: Plain language statement; BDQ: Brief demographic questionnaire; BG: Blood glucose; BGM: Blood glucose meter; GP: General practitioner; PN: Practice nurse; CDE-RN: Credentialed diabetes educator- registered nurse.

## Competing interests

JS is a member of the Accu-Check Advisory Board (Roche Diagnostics Australia). Her research group has received unrestricted educational grants from Medtronic and Sanofi Diabetes; sponsorship to host or attend educational meetings from Lilly, Medtronic, MSD, Novo Nordisk, Roche Diagnostics Australia, and Sanofi Diabetes; consultancy income from Abbott Diabetes Care, Roche Diagnostics Australia and Sanofi Diabetes. DNO, DL and JMN had various financial relationships with pharmaceutical industries outside the submitted work including consultancies, grants, lectures, educational activities and travel.

## Authors’ contributions

JF, IB, DY, and JB conceived of the study. JS and EHT advised on the psychological outcomes to be assessed and all authors advised on the content of the intervention (practice pack and participant pack). All the authors contributed to refining the study design and finalizing the protocol. JF drafted the first version of the paper. All the authors provided input to the paper and authorized the final version.

## Supplementary Material

Additional file 1 Study design. Click here for file

Additional file 2 Intervention elements. Click here for file

Additional file 3 **Glargine Titration Schedule.** Glulisine Titration Schedule. Click here for file
