# Supplementary material for: Can primary care team-based transition to insulin improve outcomes in adults with type 2 diabetes: the stepping up to insulin cluster randomized controlled trial protocol
Source: Implement Sci. 2014 Feb 14;9:20. doi: 10.1186/1748-5908-9-20 (PMC3930818; doi:10.1186/1748-5908-9-20)
Supplement: Additional file 1 — Study design. [file 1748-5908-9-20-S1.pdf]

Glargine Titration Schedule

Initial starting dose of glargine will be 10 units administered at bed-time though the insulin dose and its timing can be modified at the discretion of the medical practitioner.

Changes to insulin dosing are to be made at visits to clinic/or via telephone, or by Accu-Chek self-titration tool each 3 days for those patients deemed appropriate for this.

Fasting Glucose (average of last 3 days)	Algorithm for Insulin Glargine Titration
<4.0mmol/L	Reduce Glargine dose by 2-4 Units
4.0 -7.0mmol/L	Leave Glargine dose unchanged
7.1-8.0mmol/L	Add 2 Units Glargine
8.1-10.0mmol/L	Add 4 Units Glargine
10mmol/L or greater	Add 6 Units Glargine

Glulisine Titration Schedule

Initial starting dose of glulisine will be 4 units administered with the meal at which 2 hour post-prandial glucose levels are most elevated above the target range. The initial insulin dose can be modified at the discretion of the medical practitioner.

Changes to insulin dosing are to be made at visits to clinic/or via telephone, or by Accu-Chek self-titration tool each 3 days for those patients deemed appropriate for this.

2hr Post-Prandial Glucose (average of last 3 days.)	Algorithm for Insulin Glulisine Titration
<4.5mmol/L	Reduce Glulisine dose by 2-4 Units
4.5 -7.0 mmol/L	Leave Glulisine dose unchanged
7.0-10.0mmol/L	Add 0-2Units Glulisine (discretion of Dr)
10.0-12.0mmol/L	Add 2 Units Glulisine
12.1-14.0mmol/L	Add 4-6 Units Glulisine
>14.0mmol/L	Add 6-8 Units Glulisine (discretion of Dr)
