# Supplementary material for: Can primary care team-based transition to insulin improve outcomes in adults with type 2 diabetes: the stepping up to insulin cluster randomized controlled trial protocol
Source: Implement Sci. 2014 Feb 14;9:20. doi: 10.1186/1748-5908-9-20 (PMC3930818; doi:10.1186/1748-5908-9-20)
Supplement: Additional file 2 — Intervention elements. [file 1748-5908-9-20-S2.pdf]

Figure 1: Study design

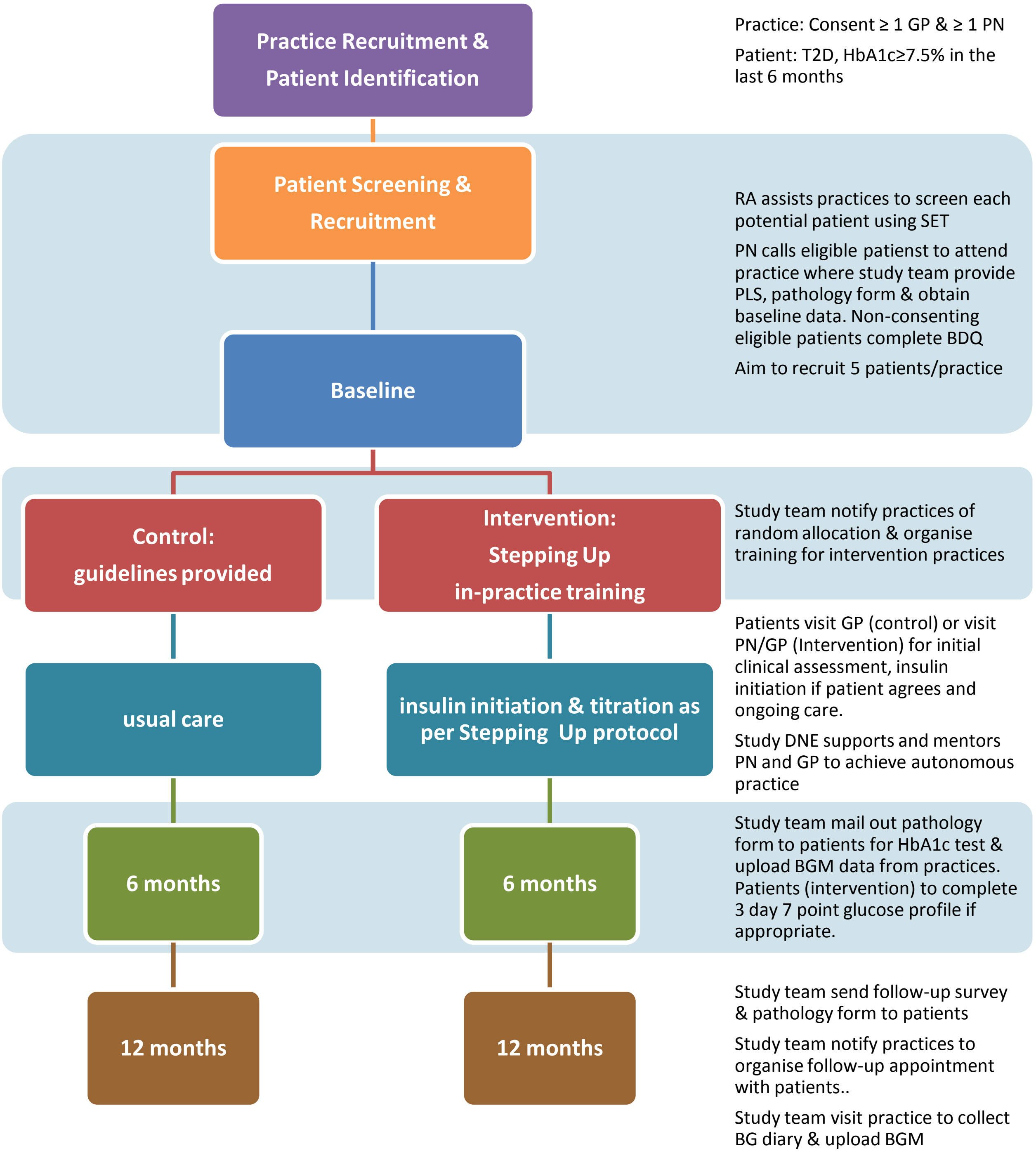

Legend

T2D= Type 2 Diabetes
RA= Research Assistant
SET = Screening Eligibility Tool
PLS = Plain Language Statement
BDQ = Brief Demographic Questionnaire
BG= Blood Glucose
BGM = Blood Glucose Meter
GP = General Practitioner
PN = Practice Nurse
DNE = Diabetes Nurse Educator
