# Supplementary material for: Can primary care team-based transition to insulin improve outcomes in adults with type 2 diabetes: the stepping up to insulin cluster randomized controlled trial protocol
Source: Implement Sci. 2014 Feb 14;9:20. doi: 10.1186/1748-5908-9-20 (PMC3930818; doi:10.1186/1748-5908-9-20)
Supplement: Additional file 3 — Glargine Titration Schedule. Glulisine Titration Schedule. [file 1748-5908-9-20-S3.pdf]

Figure 2: Intervention elements

<u>1: In-Practice Briefing and Training Visit</u>: Participating GPs and the PN in *intervention practices* will participate in an *In-Practice Briefing and Training Visit* led by the study DNE and/or GP. Case studies derived from general practice patients are used as the basis of training. At this visit a presentation is made and the **Stepping Up Program Manual** is detailed to GPs and PN. The manual includes a **Practice Pack** and a copy of the **Patient Pack.**

Issues covered in the presentation and associated with resources in the **Practice Pack** include:

- Evidence and rationale for use of insulin in T2D;
- Current clinical guidelines;
- Common patient concerns and how to deal with them;
- Motivational interviewing and goal setting strategies;
- Frequently asked questions sheet;
- Protocol and checklist for patient Day 1 and Day 2 visits with PN for discussion and commencement of insulin Glargine;
- Algorithm for simple dose adjustment based on Davies et al [1] (see Appendices);
- Templates for recording dose adjustment consultations with patients; and hands on familiarisation with insulin delivery systems.

The Patient Pack contains information and fact sheets (from Diabetes Australia - Victoria and The National Diabetes Services Scheme) supporting the use and day-to-day management of insulin in T2D; A Diary and Phone Record book for use in dose adjustment consultation with the PN.

Within the training the roles and responsibilities of GP, PN, DNE and other health professionals in the commencement of insulin are discussed, and practice staff are encouraged to identify local DNE and Endocrinologists for clinical support and referral in ongoing clinical management of patients.

<u>2: Practice and PN support structures:</u> The study DNE works as a mentor with intervention practices to *individualise and embed the model of care within their practice systems* and to support the sustainability of PNs in this enhanced role as practices become more autonomous in the work. Roles for the Study DNE include:

- Being present at initial insulin initiation visit with the first participating patient; and
- Supporting (either by face to face or phone) the GP and PN to move to independence in this process with subsequent patients at a mutually agreed time
